# Salivary Gland Toxicity of PSMA-Targeted Radioligand Therapy with ^177^Lu-PSMA and Combined ^225^Ac- and ^177^Lu-Labeled PSMA Ligands (TANDEM-PRLT) in Advanced Prostate Cancer: A Single-Center Systematic Investigation

**DOI:** 10.3390/diagnostics12081926

**Published:** 2022-08-10

**Authors:** Thomas Langbein, Harshad R. Kulkarni, Christiane Schuchardt, Dirk Mueller, Gerd Fabian Volk, Richard P. Baum

**Affiliations:** 1 Theranostics Center for Molecular Radiotherapy and Molecular Imaging, Zentralklinik Bad Berka, 99438 Bad Berka, Germany; 2 Department of Nuclear Medicine, Technical University of Munich, Klinikum Rechts der Isar, 81675 Munich, Germany; 3 BAMF Health, Grand Rapids, MI 49503, USA; 4 Department of Nuclear Medicine, University Hospital Ulm, 89081 Ulm, Germany; 5 Department of Otorhinolaryngology, Facial-Nerve-Center Jena, Center for Rare Diseases Jena, Jena University Hospital, 07743 Jena, Germany; 6 CURANOSTICUM Wiesbaden-Frankfurt, Center for Advanced Radiomolecular Precision Oncology, 65191 Wiesbaden, Germany

**Keywords:** PSMA, radioligand therapy, mCRPC, salivary gland toxicity, xerostomia

## Abstract

Purpose: PSMA-targeted radioligand therapy (PRLT) is a promising treatment option for patients with metastatic castration-resistant prostate cancer (mCRPC). However, a high uptake of the radiopharmaceutical in the salivary glands (SG) can lead to xerostomia and becomes dose-limiting for ^225^Ac-PSMA-617. This study investigated the sialotoxicity of ^177^Lu-PSMA-I&T/-617 monotherapy and co-administered ^225^Ac-PSMA-617 and ^177^Lu-PSMA-617 (Tandem-PPRLT). Methods: Three patient cohorts, that had undergone ^177^Lu-PSMA-I&T/-617 monotherapy or Tandem-PRLT, were retrospectively analyzed. In a short-term cohort (91 patients), a xerostomia assessment (CTCAE v.5.0), a standardized questionnaire (sXI), salivary gland scintigraphy (SGS), and SG SUVmax and the metabolic volume (MV) on ^68^Ga-PSMA-11-PET/CT were obtained before and after two cycles of ^177^Lu-PSMA-I&T/-617. In a long-term cohort, 40 patients were similarly examined. In a Tandem cohort, the same protocol was applied to 18 patients after one cycle of Tandem-PRLT. Results: Grade 1 xerostomia in the short-term follow-up was observed in 22 (24.2%) patients with a worsening of sXI from 7 to 8 at (*p* < 0.05). In the long-term cohort, xerostomia grades 1 to 2 occurred in 16 (40%) patients. SGS showed no significant changes, but there was a decline of the MV of all SGs. After Tandem-PRLT, 12/18 (66.7%) patients reported xerostomia grades 1 to 2, and the sXI significantly worsened from 9.5 to 14.0 (*p* = 0.005), with a significant reduction in the excretion fraction (EF) and MV of all SGs. Conclusion: ^177^Lu-PSMA-I&T/-617 causes only minor SG toxicity, while one cycle of Tandem-PRLT results in a significant SG impairment. This standardized protocol may help to objectify and quantify SG dysfunction.

## 1. Introduction

With approximately 1,276,000 new diagnoses and 359,000 deaths in 2018, prostate cancer (PC) is the second-most common and one of the deadliest cancers in men worldwide [1]. In 16% of the patients, metastases are detected within 10 years after definitive therapy, and about 3% of patients will be diagnosed with primary metastatic disease [2,3]. The 5-year survival rate drops distinctly from 98% for localized to 30% in the advanced disease [4]. After failing androgen deprivation therapy (ADT), patients develop metastatic castration-resistant prostate cancer (mCRPC), which is associated with a poor prognosis and a 5-year survival rate of 15% [4,5].The transmembrane protein (84 kDa) prostate-specific membrane antigen (PSMA) shows a distinct overexpression on PC cells, which increases with a higher Gleason score and metastatic disease [6,7,8,9]. Additionally, due to its continuous cell internalization, PSMA therefore became an excellent target for Theranostics in PC. However, physiological PSMA expression has also been detected in other organs (e.g., in the proximal renal tubule, duodenum, and parotid glands [6]). Since 2002, PSMA-targeting radiopharmaceuticals have been investigated [10], with ^68^Ga-PSMA-11 being the first PET tracer translated to human use [11,12].

PSMA-targeted radioligand therapy (PRLT) commonly uses the beta-emitter Lutetium-177 (^177^Lu) or the alpha-emitter Actinium-225 (^225^Ac). However, the first clinical endoradiotherapies were performed with ^131^I-MIP-1095, reporting significant rates of salivary gland (SG) toxicity [13]. The worldwide first Lutetium-PSMA radioligand therapy was performed in 2013 using PSMA-I&T at Zentralklinik Bad Berka. The initial results of ^177^Lu-PSMA-I&T/-617 PRLT showed promising response rates [14,15,16,17]. However, grade 1 dryness of the mouth occurred in 87% of the patients in a phase II study [18], and in a phase III trial that recently led to the FDA approval of ^177^Lu-PSMA-617, grade 1 to 2 xerostomia was observed in 38.8% of the patients [19]. Despite the high antitumor efficacy of ^225^Ac-PSMA-617 PRLT, the severity of xerostomia was much higher and became even the dose-limiting toxicity factor [20,21,22,23]. Therefore, Tandem-PRLT with the coadministration of ^177^Lu-PSMA-617 and lower activities of ^225^Ac-PSMA-617 was introduced, demonstrating comparable initial response rates [24]. Still, little is known about the underlying mechanism of the SG uptake of PSMA-targeting radiopharmaceuticals. In a preclinical study, ^177^Lu-PSMA-617 accumulation in the SG was considered partly PSMA-specific, with a high nonspecific uptake fraction [25], while other authors suggested a predominantly non-PSMA-mediated uptake [26].

The impact on quality of life due to severe xerostomia is known from, e.g., patients after radiotherapy for head and neck cancer or after radioiodine therapy for thyroid cancer [27,28,29]. Salivary gland scintigraphy (SGS) has been previously established to assess the post-therapeutic SG dysfunction [30].

In this retrospective analysis, we aimed to systematically investigate and objectify the short-term and long-term SG toxicity of ^177^Lu-PSMA-I&T/-617 PRLT in mCRPC patients by using a standardized protocol. For quantification of the SG dysfunction, a validated questionnaire, the SGS and PSMA-PET/CT parameters were used and predictors for SG hypofunction after PRLT were studied. In a sub-investigation, the early SG dysfunction of Tandem-PRLT was examined for comparison with the data of ^225^Ac-PSMA-617 monotherapy. 

## 2. Material and Methods

### 2.1. Study Design

Due to the retrospective data acquisition and analysis of this investigation, ethical review and approval were waived by the institutional review board. All patients gave their written informed consent. All procedures were in compliance with The German Medicinal Products Act, AMG §13 2b, the conditions of the Declaration of Helsinki article 37 “Unproven interventions in clinical practice“, and the responsible regulatory body (Government of Thuringia).

The institutional eligibility criteria for PRLT have been published before [14,31] and are outlined in Supplementary Table S1. Patients with mCRPC and prior taxane-based chemotherapy, ineligibility for chemotherapy, or explicit refusal of chemotherapy were suitable. A pretherapeutic ^68^Ga-PSMA-11-PET scan had confirmed intense PSMA expression of the tumor lesions. The dosing of ^177^Lu-177-PSMA-I&T/-617 and ^225^Ac-PSMA-617 PRLT were patient-individualized based on, e.g., the tumor burden, renal function, bone marrow reserve, and pretreatments. Tandem-PRLT with the combined administration of ^225^Ac- and ^177^Lu-PSMA-617 was indicated in patients with a critically high tumor load or after patients failed or became refractory to ^177^Lu-PSMA-I&T/-617 monotherapy, respectively [24]. The clinical management and follow-up of PRLT were based on national and international consensus recommendations and guidelines, respectively [32,33]. The radiolabeling and administration of ^68^Ga-PSMA-11, ^177^Lu-PSMA-I&T/-PSMA-617, and ^225^Ac-PSMA-617 were performed as previously published [12,15,21,34].

### 2.2. Patient Population

**Figure 1:**
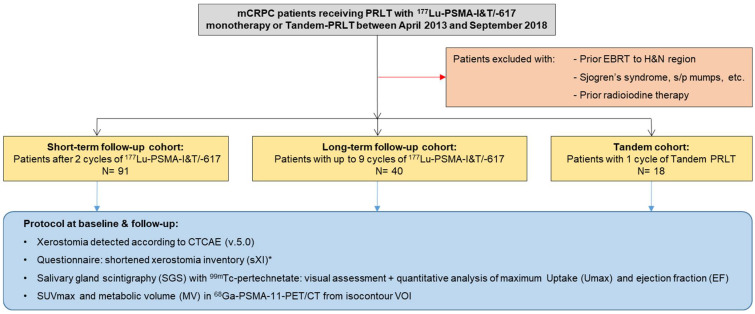
Patient cohorts and study workflow. * For the long-term cohort, data of sXI were only available at follow-up. EBRT = external beam radiotherapy; H&N = head and neck; PRLT = PSMA-targeted radioligand therapy.

From the institutional database, three cohorts of patients receiving their first cycle of either ^177^Lu-PSMA-I&T/-617 monotherapy or Tandem-PRLT between April 2013 and September 2018 were identified. The exclusion criteria were prior external radiotherapy to the head and neck region; comorbidities known to affect the baseline salivary gland function (e.g., Sjögren’s syndrome, mumps, etc.); or prior radioiodine therapy (Figure 1).

In the first cohort (short-term cohort), 91 patients were included who underwent 2 cycles of ^177^Lu-PSMA-I&T/-617. The age at first cycle, initial Gleason score, tumor distribution on baseline PSMA-PET/CT, prior mCRPC treatment lines, and the cumulative dose of ^177^Lu-PSMA-I&T/-617 were acquired. Patients were categorized based on their visually assessed tumor burden on the baseline PSMA-PET/CT (low, moderate, or high; Supplementary Figure S1), as previously described [35]. Additionally, three evenly distributed, age-based subgroups were created (<65 years, 65–72 years, and >72 years at PRLT initiation) in this cohort. In a second cohort (long-term cohort), 40 mCRPC patients were included for analysis on a long-term follow-up after ^177^Lu-PSMA-I&T/-617 monotherapy. Similar to the short-term cohort, the baseline parameters and the cumulative dose of ^177^Lu-PSMA-I&T/-617 were obtained. The time between the first PRLT cycle and last follow-up was calculated. Three evenly distributed subgroups based on the cumulatively administered activity of ^177^Lu-PSMA-I&T/-617 were created (<30 GBq, 30–40 GBq, and >40 GBq).

The third cohort consisted of 18 patients with available follow-up data after one cycle of Tandem-PRLT (Tandem cohort). Similarly, the baseline parameters and cumulative administered activity of ^177^Lu-PSMA-I&T/-617 alone or of ^225^Ac- and ^177^Lu-PSMA-617 during Tandem-PRLT were collected.

### 2.3. Assessment of Salivary Gland Function

**Figure 2:**
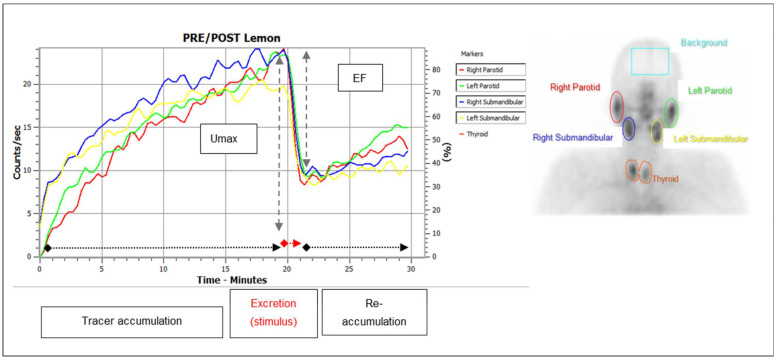
Normal time–activity curves on salivary gland scintigraphy (**left**) detected by regions of interest (ROI) over the parotid and submandibular glands (**right**). Calculation of the excretion fraction (EF): U_12–14_/U_18–20_ = tracer uptake averaged from 12–14/18–20 min after tracer injection.

The workflow based on a protocol of parameters obtained from patient charts and diagnostic procedures in all three patient cohorts is outlined in Figure 1. Xerostomia was documented according to the CTCAE (version 5.0) and results of a validated questionnaire—the shortened xerostomia inventory (sXI, five xerostomia-related items, summated score between 5 and 25 points; Supplementary Table S2) [36,37]-were acquired. Dynamic salivary gland scintigraphy (SGS) was performed as previously described, using 70 ± 10 MBq ^99m^Tc-pertechnetate [38,39]. To avoid premature stimulation of the SG, patients were asked to fast for at least 6 h prior to the SGS, brushing of their teeth, and additional potential salivary stimulators, e.g., chewing gum, were not allowed. A standardized excretion stimulus of 5 mL of lemon juice was administered orally 20 min after tracer injection. First, SGS were visually assessed based on established stages of SG dysfunction [30]. The SG function was graded from stage 0 (normal) to stage 3 (severely impaired) based on the qualitatively evaluated tracer uptake and excretion (Supplementary Table S3). Subsequently, the maximum tracer uptake (Umax) and the excretion fraction (EF) of the parotid (PG) and submandibular glands (SMG) were calculated using manually drawn regions of interest (ROI) (Figure 2). From PSMA-PET/CT (acquired 60–80 min after i.v. injection of 1.8–2.2 MBq ^68^Ga-68-PSMA-11 per kg bodyweight), the SUVmax and the metabolic volume (MV) of PG and SMG were acquired. For this purpose, standardized, 3-dimensional volumes of interest (VOIs) with an isocontour threshold of 20% of the SUVmax were used, as previously described [40,41]. To investigate influences on the baseline SG function and predictors for the SG toxicity of ^177^Lu-PSMA-I&T/-617, age at PRLT initiation, previous taxane-based chemotherapy, tumor burden on the baseline PSMA PET/CT, and the cumulatively administered activity of ^177^Lu-PSMA-I&T/-617 were assessed as independent factors.

### 2.4. Statistical Analysis

The results are reported as the frequency (percentage), median with 25–75th percentiles (IQR), median with range, mean ± standard deviation (SD), ranges, and/or 95% confidence intervals, as noted accordingly. In the case of normal distribution, a Student’s *t*-test and univariate analysis of variance (one-way ANOVA) with a post hoc analysis using Tukey’s HSD test were applied. In the case of non-normal distribution, the Wilcoxon’s signed ranks test, Mann–Whitney *U* test, and Kruskal–Wallis H test were performed.

For the univariate analysis, the mean values of all 4 investigated SG were calculated; a multivariate analysis of variance (one-way MANOVA) was conducted with a post hoc analysis using the Bonferroni procedure and the Scheffe test. A correlation analysis with subsequent linear regression was performed using Pearson’s or Spearman’s correlation, as outlined. For each test, a *p*-value of less than 0.05 was considered statistically significant. Statistical analysis was performed using SPSS Statistics, version 24 (IBM Co., Armonk, NY, USA).

## 3. Results

### 3.1. Short-Term Follow-Up Cohort

Characteristics of the 91 patients of the short-term cohort (median age: 68 years; range 46–90) are outlined in Table 1. All patients had been administered two cycles of ^177^Lu-PSMA-I&T/-617 (median 14.3 GBq, range 9.5–20.2), with a median follow-up of 2.3 months (range 1.9–2.7). Forty-four (48.4%) patients of this cohort had been pretreated with taxane-based chemotherapy.

Grade 1 xerostomia was reported in 13 (14.3%) patients at the baseline and in 22 (24.2%) patients after two cycles of Lu-177 PSMA-I&T/-617 (*p* < 0.01), with a correlated significant increase of the median sXI-score from 7 (IQR 5.3–9) before to 8 (IQR 6.3–11) after PRLT (*p* < 0.05). In addition, a moderate correlation of xerostomia symptoms and the sXI-score was found during follow-up (r = 0.43, *p* < 0.01).

Visual SGS grading before and after PRLT showed no significant difference (median stage 0 (range 0–2) vs. median stage 0 (range 0–2), *p* = 0.34). Of note, a mildly impaired SG function (stage 1 to 2) was detectable already at the baseline in 33 (36.3%) patients. A quantitative assessment confirmed no significant changes of Umax and EF after PRLT (Table 2). However, patients that reported dryness of mouth after PRLT showed significantly lower Umax and EF values of the PG at the follow-up than did asymptomatic patients (mean Umax: 0.27 ± 0.09(SD) vs. 0.35 ± 0.12(SD), *p* < 0.01; mean EF: 46.0% ± 13.8(SD) vs. 56.2% ± 11.3(SD), *p* < 0.01). No similar correlation was detectable for the SMG. The SUVmax of both the PG and SMG showed no significant changes, whereas the MV of all four SG declined significantly (*p* < 0.001, Table 2).

### 3.2. Long-Term Follow-Up Cohort

In this cohort, 40 patients were included, receiving a median of 5.5 (range 2–9) cycles of PRLT and a median cumulative activity of 35.3 GBq (range 9.9–61.8) ^177^Lu-PSMA-I&T/-617. The median follow up was 22.7 months (IQR 16.4–30.2). Nineteen (47.5%) patients had prior taxane-based chemotherapy. The patient details are outlined in Table 1.

From the baseline to follow-up, xerostomia became more frequent (grade 1 in 2 (5%) patients at baseline, grade 1 in 15 (37.5%) patients, and grade 2 in 1 (2.5%) patient at follow-up; *p* < 0.001). No grade 3 xerostomia occurred. The data of the sXI questionnaires were available only at the follow-up, showing a moderate but significant correlation to the subjective dryness of mouth (r = 0.41, *p* < 0.05).

**Figure 3:**
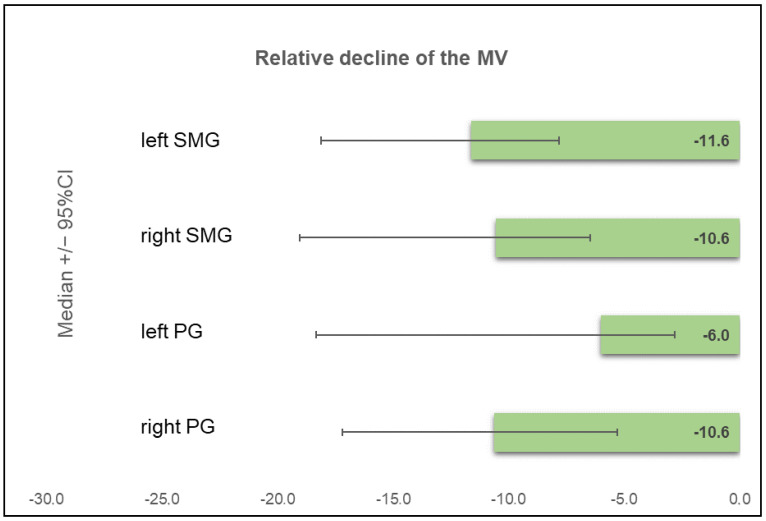
Relative decline of the metabolic volume (MV) of all 4 salivary glands during follow-up PET/CT compared to the baseline in the long-term cohort. (PG = parotid gland; SMG = submandibular gland).

In the visual grading, no significant changes were found on SGS after PRLT (stage 0 in 16 (40%) patients, stage 1 in 16 (40%) patients, and stage 2 in 8 (20%) patients at the baseline; stage 0 in 16 (40%) patients, stage 1 in 20 (50%) patients, and stage 2 in 4 (10%) patients at the follow-up; *p* = 0.63). A comparison of the Umax and EF of all SG confirmed no significant changes before and after PRLT (Table 3). PRLT-emergent xerostomia was also significantly correlated with a lower EF of the PG in this cohort (r = −0.50, *p* < 0.01). In patients without dryness of the mouth, the EF of the PG at follow-up were significantly higher than in patients with xerostomia (EF 54.0% ± 11.7 (SD) vs. 40.0% ± 16.4 (SD); *p* < 0.001). No correlation of the Umax to the subjective complaints was found, and no similar correlation could be demonstrated for the submandibular glands. There was a trend towards a lower SUVmax of all SGs after PRLT; however, without statistical significance (Table 3), PSMA-PET/CT showed a significant decline of the MV of all SGs (*p* < 0.001), with a median of −6.0% (95%CI: −18.3 to −2.8%) to −11.6% (95%CI: −18.1 to −7.8%) (Figure 3).

### 3.3. Tandem-Cohort PRLT

Eighteen patients were identified for analysis that had been administered a median activity of 4.0 MBq (range 2.0–7.0) ^225^Ac-225 PSMA-617 and 4.25 GBq ^177^Lu-PSMA-617 (range 3.6–7.2 GBq) for Tandem-PRLT. Seven (38.9%) patients had been pretreated with taxane-based chemotherapy, and fourteen (77.8%) patients had been treated with ^177^Lu-PSMA-I&T/-617 monotherapy before Tandem-PRLT was initiated. Further patient details are shown in Table 1.

One-third (6/18) of the patients reported grade 1 xerostomia at the baseline, while, after one cycle of Tandem-PRLT, xerostomia grade 1 in 10/18 patients and grade 2 in 2/18 patients was observed (*p* = 0.001), and 6/18 patients did not report any xerostomia at the follow-up. There was no patient-requested treatment discontinuation. The sXI-score increased significantly from 9.5 (95%CI: 7.0–14.2) before to 14.0 (95%CI: 11.5–19.6) after Tandem-PRLT (*p* = 0.005). The stage of xerostomia at the baseline according to SGS was 0 in 10/18 patients, 1 in 6/18 patients, and 2 in 2/18 patients, while, at the follow-up, stage 0 was noted in 1/18 patients, stage 1 in 8/18 patients, and stage 2 in 9/18 patients (*p* < 0.001).

**Figure 4:**
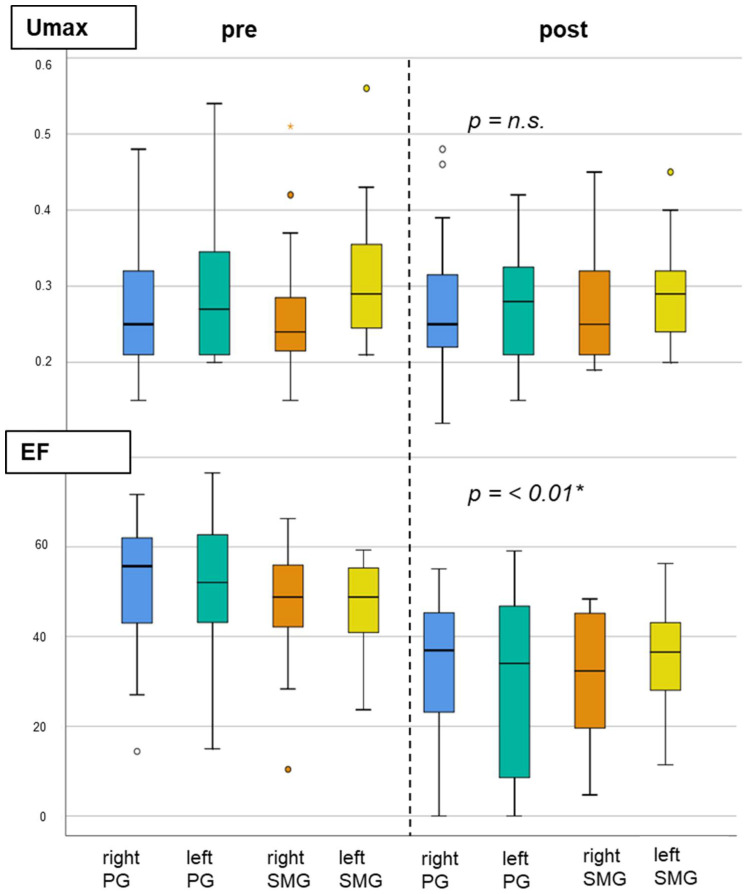
Quantitative analysis of the salivary gland scintigraphy before and after 1 cycle of Tandem-PRLT. While the Umax of all salivary glands showed no significant changes (**top**), the EF declined significantly in all salivary glands (**bottom**). PG = parotid gland; SMG = submandibular gland. * Wilcoxon test.

The Umax of all four SG showed no significant changes at the follow-up, whereas the EF of all SG declined significantly (*p* < 0.01; Figure 4). The SUVmax showed a nonsignificant trend towards lower values after treatment (Supplementary Table S4), while the MV of all four SG decreased significantly (*p* < 0.05; right PG: median −12.2% (95%CI: −21.6 to −6.1); left PG: median –7.8% (95%CI: −20.5 to −0.1); right SMG: median −8.7% (95%CI: −16.3 to 0.4); left SMG: median –6.5% (95%CI: −12.1 to 1.1)).

### 3.4. Co-Factors for Salivary Gland Toxicity

**Figure 5:**
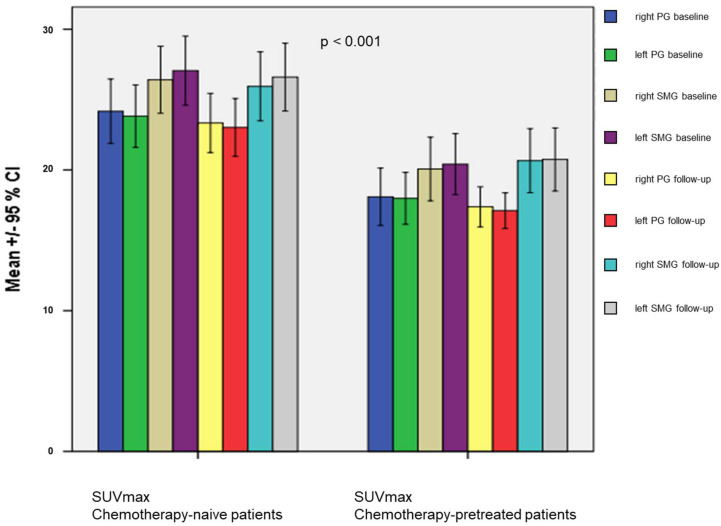
SUVmax of the salivary glands at the baseline and follow-up PSMA PET/CT. PG = parotid gland; SMG = submandibular gland.

Significantly higher Umax values of the SG both during the baseline and on the follow-up SGS were observed in older patients in the subgroup analysis (*p* < 0.001). However, no influence of the patient’s age on EF, the PSMA-PET/CT-based parameters and reported xerostomia was found. Chemotherapy pretreated patients in the short-term cohort showed significantly lower SUVmax of all SG both before and after PRLT (baseline median SUVmax: 24.7 vs. 18.2; follow-up median SUVmax: 24.0 vs. 18.8, *p* < 0.001; Figure 5). However, there was no significant differences between chemotherapy-pretreated and chemotherapy-naïve patients in terms of the xerostomia, MV, and SGS parameters. The SUVmax of all SG showed a significant correlation with the tumor burden at the baseline PSMA PET/CT, with lower SUVmax in patients with higher tumor loads (*p* < 0.001; Kruskal–Wallis test). No significant differences between the three subgroups stratified by tumor burden in terms of the reported xerostomia, sXI-scores, SGS parameter, and the MV at the baseline and follow-up were found (Supplementary Figure S2).

In the long-term cohort, patients with higher cumulative administered activity showed lower SUVmax at the follow-up PSMA-PET/CT (*p* < 0.05; Kruskal–Wallis test). However, no influence of the cumulatively administered ^177^Lu-PSMA-I&T/-617 on the MV, Umax, EF, the reported xerostomia, and the sXI-scores was found at the follow-up (Supplementary Figure S3).

## 4. Discussion

In this single-center, retrospective investigation of SG toxicity of ^177^Lu-PSMA-I&T/-617 PRLT in 91 patients with short-term and in 40 patients with long-term follow-up, only mild-to-moderate subjective and objective SG dysfunction was observed in only a minority of the patients. In contrast, only one cycle of Tandem-PRLT caused a much more distinct salivary gland impairment.

Until today, salivary gland toxicity, especially of ^225^Ac-PSMA-I&T/-617 PRLT, remains an unsolved clinical issue, while the data on sialotoxicity after ^177^Lu-PSMA-I&T/-617 appears heterogeneous. In early retrospective studies, grade 1 to 2 xerostomia was reported in 8–24% of cases [15,16,17], while, in a phase II trial, xerostomia occurred in 87% of the patients [18] and in 38.8% of the patients in a phase III trial [19]. In our study, xerostomia grade 1 was observed in 24.2% of the patients after two cycles of ^177^Lu-PSMA-I&T/-617, while 40% of the patients reported grade 1 to 2 xerostomia during the long-term follow-up (maximum follow-up of 52 months), receiving up to 61.8 GBq. Previously, a transient character of xerostomia has been described by several authors, usually resolving 3 months after therapy [14,17]. Therefore, the time point of interviewing patients for dryness of mouth appears crucial. For the quantification of xerostomia, we used a validated questionnaire with a good symptomatic correlation in short-term and long-term follow-up.

SGS has been previously used to objectify SG dysfunction after, e.g., external radiotherapy [42,43,44] and radioiodine therapy [30,45], since a good correlation to xerostomia and saliva flow rates has been shown, and SG impairments can be detected already at early stages [39,46]. While, in our study, no intra-patient changes on SGS after two cycles of ^177^Lu-PSMA-I&T/-617 were found, patients with xerostomia at follow-up showed lower values of the PG for both Umax and EF. These findings are in line with the data after radioiodine therapy in patients with differentiated thyroid cancer, in which a bilateral PG dysfunction was the most common condition [47]. Already, after low doses of 0.4–0.6 GBq ^131^I, a 14% decline of the Umax was observed, while, after 24 GBq of ^131^I, the Umax decreased by 90% [38]. The SGS in head and neck cancer patients after external radiotherapy showed a 50% decline of the PG EF [44]. Another study reported a significant decrease of the tracer uptake of the PG after radiotherapy, with an EF decline from 44.7% to 18.7% post-therapeutically [42].

At the early follow-up, no significant changes of the SUVmax were found in our cohort, whereas a significant decline of the MV was observed, confirmed in the long-term cohort, in which the MV declined between a median 6.0 and 11.6%. Lower SUVmax were detected at the long-term follow-up; however, this was without statistical significance, possibly due to the low patient number. In addition, patients who received a higher cumulative activity showed a significantly lower SUVmax at the follow-up. Comparable results were previously published, with a decline of 20% in the PG volume and of 9.8% in the SMG and a decrease in the SUVmax by 6% for the PG and 10.5% for the SMG after two to three cycles of ^177^Lu-PSMA-617 [40]. Therefore, SUVmax and MV might act as early indicators of SG toxicity. However, these findings need to be confirmed on a larger scale. Zhao et al. described the ^68^Ga-PSMA-11 PET/CT parameters for SG as a helpful supplement to SGS in patients with different degrees of SG dysfunction due to either Sjögren’s syndrome, radiotherapy for head and neck cancer, or after surgery of the SGs [41].

However, some limitations of using PSMA PET/CT for evaluation of the SG function must be stated. Both a high intra- and interindividual variability of the SG SUVmax were demonstrated [48], which could be confirmed in our study. Moreover, PSMA ligand uptake cannot assess the excretion function of the SGs and might be affected by the tumor sink effect [49,50]. Consequently, a significant negative correlation of the SUVmax of all SGs to the tumor burden both pre- and post-therapeutically was found in our data.

Overall, the parameters of SG function investigated in this study suggest a combined use of PSMA-PET/CT and SGS for the screening of early SG toxicity. While the tracer uptake (SUVmax on PET/CT and Umax on SGS) showed a lower correlation to the clinical symptoms, changes of the MV on PET/CT and the EF on SGS might be useful, sensitive surrogates of SG dysfunction.

No influence of prior chemotherapy on the SGS parameter or the subjective parameter of hyposalivation was detected. However, significantly lower SUVmax were observed in chemotherapy-pretreated patients. Another study comparing PRLT in chemotherapy-naïve and chemotherapy-pretreated patients found no difference in the frequency of xerostomia between both groups [51].

A median absorbed dose to SG of between 0.5 and 1.4 Gy/GBq, administering ^177^Lu-PSMA-I&T/-617, has been published [14,15,52,53], which translates to an estimated mean absorbed dose of 3.0–8.4 Gy per cycle of 6.0 GBq or of 3.8–10.5 Gy/cycle of 7.5 GBq ^177^Lu-PSMA-I&T/-617, respectively.

A recent study comparing the biodistribution and dosimetry data of ^177^Lu-PSMA-I&T and ^177^Lu-PSMA-617 in 138 mCRPC patients demonstrated no significant difference in the absorbed SG dose of both PSMA targeting small molecules [54].

The threshold of the maximum tolerable SG dose of external radiotherapy is still a subject of discussion, with 26–45 Gy being proposed for the PG [55,56]. Based on this, Scarpa et al. estimated a maximum tolerable activity of ^177^Lu-PSMA of 60 GBq, assuming a mean dose of 0.5 Gy/GBq for the SG [40]. However, concerning the limited radiobiological comparability of external irradiation and endoradiotherapies, such estimates should be undertaken with caution.

First, the clinical data for ^225^Ac-PSMA-617 in mCRPC patients were published by the Heidelberg group, demonstrating encouraging responses, even after failing to find ^177^Lu-PSMA-617 [20,22]. However, 4 of the initial 40 patients discontinued treatment because of severe xerostomia or loss of taste, despite the initial response. Further, 15 patients with partial remission eventually stopped treatment due to xerostomia. Another study reported severe dryness of mouth after ^225^Ac-PSMA-617 that led to treatment discontinuation in about one-third of the patients [23]. Sathekge et al. suggested a dose deescalating scheme of ^225^Ac-PSMA-617 [57]. In 73 mCRPC patients, xerostomia grade 1 to 2 was reported in 85% of the patients, while no patient showed a grade 3 disease, and no treatment discontinuation occurred. In 12/18 patients (66.7%), our study showed grade 1 to 2 dryness of mouth after one cycle of Tandem-PRLT, which correlated with a significant increase in the sXI-score. On the other hand, one-third of the patients did not notice any dryness of mouth, and no grade 3 xerostomia or therapy discontinuation emerged. However, the limited comparability of the published studies to our cohort must be underlined in terms of, e.g., applied activities, cycles of ^225^Ac-PSMA-617, and prior treatments.

Khreish et al. investigated the concept of co-administering lower activities ^225^Ac-PSMA-617 (mean 5.3 MBq) and ^177^Lu-PSMA-617 (mean 6.9 GBq) in 20 mCRPC patients and found comparable results. In 8/20 patients, they found grade 1 and 5/20 patients grade 2 xerostomia. No grade 3 dryness of mouth and no treatment discontinuation was observed [58].

In another study, SGS in 11 patients after up to four cycles of ^225^Ac-PSMA-617 showed a distinct decline of both the Umax and the EF [37]. In contrast, in our study, we found no changes of the Umax after Tandem-PRLT. However, the EF of all SG declined significantly.

A reason for xerostomia after ^225^Ac-PSMA-617 PRLT might be a severe duct stenosis or obstruction, as it has been described before in patients after radioiodine therapy and external radiotherapy [42,59]. Consequently, the patients showed a response to sialendoscopic duct dilatation and saline irrigation [37].

Preventing the SG uptake of PSMA-targeting radiopharmaceuticals is still a clinical unmet need, and different approaches were investigated in the past [60,61]. Additionally, external cooling [62,63], orally administered monosodium glutamate [64,65], and recently, high-dose botulinum toxin injections for radioprotection of the PG and SMG were clinically tested [66,67], with preliminary encouraging data on the effectiveness.

The limitations of our study included its retrospective and single-center design, resulting in a potential selection bias of the cases and limited statistical power of the analysis, especially for the subgroup investigations in the short-term and long-term cohorts and the analysis of the Tandem cohort. However, our data might encourage future prospective, multi-institutional studies to investigate our findings on a larger scale.

## 5. Conclusions

Salivary gland dysfunction after ^177^Lu-PSMA-I&T/-617 PRLT has minor clinical relevance, both subjectively and objectively. Even after high cumulative activities, only mild-to-moderate dryness of mouth occurs in a minority of the patients. The prevalence of xerostomia appears to be significantly lower than the historical controls after external radiotherapy, radioiodine therapy, and especially after PRLT with ^225^Ac-PSMA-617. A validated questionnaire on xerostomia, salivary gland scintigraphy, and PSMA-PET/CT parameters can help to objectify, standardize, and quantify the SG toxicity of PRLT. A decrease of the excretion fraction on SGS and of the metabolic volume on PSMA PET/CT can be early indicators of SG impairment. The co-administration of lower doses of ^225^Ac in combination with ^177^Lu-PSMA-617 (Tandem concept) can decrease severe xerostomia after PRLT with alpha-emitters.

## Figures and Tables

**Table 1:** Characteristics of the 3 investigated patient cohorts. PRLT = radioligand therapy: IQR = interquartile range.

Characteristic	Short-Term Cohort; *n* = 91	Long-Term Cohort; *n* = 40	Tandem Cohort; *n* = 18
Age at first cycle of PRLT (median; range); years	68 (46–90)	68 (50–90)	65 (52–82)
Initial Gleason score (median; range)	8 (6–10)	8 (5–10)	8 (6–10)
Metastases at baseline PET/CT			
Bone metastases	77 (84.6%)	35 (87.5%)	17 (94.4%)
Lymph node metastases	73 (80.2%)	33 (82.5%)	15 (83.3%)
Visceral metastases	23 (25.3%)	11 (27.5%)	5 (27.8%)
Tumor burden based on base PSMA PET/CT			
Low	34 (37.4%)	15 (37.5%)	0 (0%)
Moderate	30 (33.0%)	13 (32.5%)	5 (27.8%)
High	27 (29.7%)	12 (30.0%)	13 (72.2%)
mCRPC pretreatments			
Chemotherapy	44 (48.4%)	19 (47.5%)	7 (38.9%)
Docetaxel	40 (44.0%)	19 (47.5%)	6 (33.3%)
Cabazitaxel	16 (17.6%)	4 (10.0%)	3 (16.7%)
androgen receptor axis-targeted agents	56 (61.5%)	32 (80.0%)	14 (77.8%)
Enzalutamide	45 (49.5%)	22 (55.0%)	11 (61.1%)
Abiraterone	37 (40.7%)	23 (57.5%)	10 (55.6%)
Prior ^177^Lu-PSMA-I&T/-617 monotherapy	n/a	n/a	14 (77.8%)
Supportive treatments during PRLT			
Bisphosphonates	27 (29.7%)	14 (35.0%)	7 (38.9%)
Denosumab	24 (26.4%)	9 (22.5%)	3 (16.7%)
Cumulative administered ^177^Lu-PSMA-I&T/-617 (median; range); GBq	14.3 (9.5–20.2)	35.3 (9.9–61.8)	
Administered ^225^Ac-PSMA-617 (median, range); MBq	n/a	n/a	4.0 (2.0–7.0)
Follow-up (median; IQR); months	2.3 (1.9–2.7)	22.7 (16.4–30.2)	2.5 (2.0–3.2)
Total cycles of 177Lu-PSMA-I&T/-617 applied (median, range)	2	5.5 (2–9)	n/a

**Table 2:** top: Quantitative results of the salivary gland scintigraphy of the short-term cohort at the baseline and follow-up: No significant differences of the maximum tracer uptake (Umax) and the excretion fraction (EF) were observed in all salivary glands. (PG = parotid gland; SMG = submandibular gland). Umax = percentage of the injected tracer activity; EF = percentage of Umax). bottom: Salivary gland parameters determined by ^68^Ga-PSMA-11 PET/CT at the baseline and follow-up: A significant decline of the MV of all 4 SG was observed. MV = metabolic volume.

	Umax						p *	EF						p *
	Baseline			Follow-Up				Baseline			Follow-Up			
	Mean	Min	Max	Mean	Min	Max		Mean	Min	Max	Mean	Min	Max	
right PG	0.32	0.11	0.73	0.31	0.12	0.63	*n.s.*	57.9	2.4	88.8	54.9	13.9	83.5	*n.s.*
left PG	0.35	0.10	0.82	0.34	0.12	0.73	*n.s.*	57.1	23.2	82.7	52.5	5.7	73.9	*n.s.*
right SMG	0.31	0.15	0.84	0.32	0.10	0.85	*n.s.*	49.9	24.9	67.9	49.1	19.7	67.7	*n.s.*
left SMG	0.33	0.13	0.67	0.33	0.10	0.91	*n.s.*	48.8	20.7	70.1	47.5	6.1	68.4	*n.s.*
	**SUVmax**						p *	**MV** (**cm^3^**)						p *
	**Baseline**			**Follow-Up**				**Baseline**			**Follow-Up**			
	Mean	Min	Max	Mean	Min	Max		Mean	Min	Max	Mean	Min	Max	
right PG	21.3	5.4	41.9	20.5	9.3	37.0	*n.s.*	36.7	11.2	61.0	33.2	3.1	57.1	*<0.001*
left PG	21.1	7.7	38.6	20.2	8.3	37.3	*n.s.*	37.1	22.7	60.6	33.8	17.4	59.5	*<0.001*
right SMG	23.2	10.1	44.6	23.4	9.8	52.1	*n.s.*	13.0	2.7	24.2	11.9	3.6	20.1	*<0.001*
left SMG	23.8	10.1	49.8	23.8	9.3	45.3	*n.s.*	13.0	7.6	26.6	11.9	5.1	19.8	*<0.001*

* Wilcoxon test.

**Table 3:** top: Results of the salivary gland scintigraphy of the long-term cohort at the baseline and follow-up: No significant differences of Umax and EF were observed in all salivary glands. (PG = parotid gland; SMG = submandibular gland). Umax = percentage of the injected tracer activity. EF = percentage of Umax. bottom: Salivary gland parameters determined by ^68^Ga-PSMA-11 PET/CT at the baseline and follow-up. A significant decline of the MV of all 4 SG was observed. MV = metabolic volume.

	Umax						p *	Ef						p *
	Baseline			Follow-Up				Baseline			Follow-Up			
	Mean	Min	Max	Mean	Min	Max		Mean	Min	Max	Mean	Min	Max	
right PG	0.29	0.12	0.50	0.35	0.13	0.70	*n.s.*	53.1	27.2	75.1	48.5	1.9	72.2	*n.s.*
left PG	0.35	0.15	0.82	0.36	0.12	0.66	*n.s.*	52.6	36.3	72.1	48.8	8.0	71.1	*n.s.*
right SMG	0.32	0.20	0.48	0.34	0.17	0.51	*n.s.*	45.5	15.7	67.0	44.6	19.7	63.7	*n.s.*
left SMG	0.34	0.22	0.58	0.35	0.19	0.56	*n.s.*	45.3	34.6	62.6	46.7	16.1	65.1	*n.s.*
	**SUVmax**						p *	**MV (cm^3^)**						p *
	**Baseline**			**Follow-Up**				**Baseline**			**Follow-Up**			
	Mean	Min	Max	Mean	Min	Max		Mean	Min	Max	Mean	Min	Max	
right PG	20.0	5.4	37.3	18.6	7.1	38.2	*n.s.*	40.5	23.6	60.7	34.5	16.5	56.4	*<0.001*
left PG	20.1	7.6	38.6	18.0	4.9	35.6	*n.s.*	38.9	5.7	60.7	33.9	5.9	55.9	*<0.001*
right SMG	21.3	9.8	39.4	20.6	10.8	46.9	*n.s.*	14.1	9.3	27.4	11.9	7.2	20.2	*<0.001*
left SMG	21.6	10.5	38.9	21.0	9.2	46.5	*n.s.*	14.1	7.6	28.2	11.9	6.6	22.5	*<0.001*

* Wilcoxon test.

## Data Availability

The data presented in this study are available on request from the corresponding author. The data are not publicly available due to privacy restrictions.

## Supplementary Materials

The following supporting information can be downloaded at: https://www.mdpi.com/article/10.3390/diagnostics12081926/s1, Figures S1–S3; Tables S1–S4.
