# Supplementary material for: Salivary Gland Toxicity of PSMA-Targeted Radioligand Therapy with 177Lu-PSMA and Combined 225Ac- and 177Lu-Labeled PSMA Ligands (TANDEM-PRLT) in Advanced Prostate Cancer: A Single-Center Systematic Investigation
Source: Diagnostics (Basel). 2022 Aug 10;12(8):1926. doi: 10.3390/diagnostics12081926 (PMC9406477; doi:10.3390/diagnostics12081926)
Supplement: Supplementary file 1 [file diagnostics-12-01926-s001.zip › diagnostics-1847188-supplementary.pdf]

SUPPLEMENTARY MATERIAL

**Disease specific**	- histologically confirmed prostate carcinoma<br>- castration resistance<br>- non-resectable metastases<br>- disease progression under guideline-based therapy<br>- proven PSMA expression by PSMA-PET/CT imaging
**Progression after chemotherapy (a or b or c must apply):**	a.) s.p. taxane-based chemotherapy<br>b.) unsuitable for chemotherapy<br>c.) refusal of chemotherapy
**Laboratory findings**	- sufficient bone marrow reserve (hemoglobin ≥5.5 mmol/l; leukocyte count >3.000/µl; thrombocyte count >75.000/µl)<br>- sufficient organ function (creatinine < 2 times the upper standard limit; AST or ALT below 5 times the upper standard limit)
**Other**	- ECOG PS 0 or 1/Karnofsky performance index >60%<br>- more than a 6 week-interval to the last potentially myelosuppressive therapy<br>- written informed consent of the patient about an individual character of the treatment (a sample of the information sheet is given in the Supplements section)

Supplementary Table S1 Institutional eligibility criteria for radioligand therapy AST=serum aspartate transaminase; ALT= serum alanine transaminase; ECOG PS = Eastern Cooperative Oncology Group Performance Status.

question/statement	answer possibilities/points
**My mouth feels dry**	*"never"* = 1
**I have difficulty in eating dry foods**	*"hardly ever"* = 2
**My mouth feels dry when eating a meal**	*"occasionally"*= 3
**I have difficulties swallowing certain foods**	*"frequently"* = 4
**My lips feel dry**	*"always"* = 5

Supplementary Table S2 Adapted version of the validated shortened xerostomia inventory (sXI) to obtain patient-reported outcome measures (PROM) for quantification of mouth dryness

stage of salivary gland function	uptake	excretion
Stage 0 – normal	normal	normal
Stage 1 – mildly impaired	mildly reduced	normal – mildly reduced
Stage 2 – moderately impaired	reduced	reduced
Stage 3 – severely impaired	severely reduced	no significant excretion

Supplementary Table S3 Visual grading of the salivary gland scintigraphy adopted from Solans *et al.* [30]

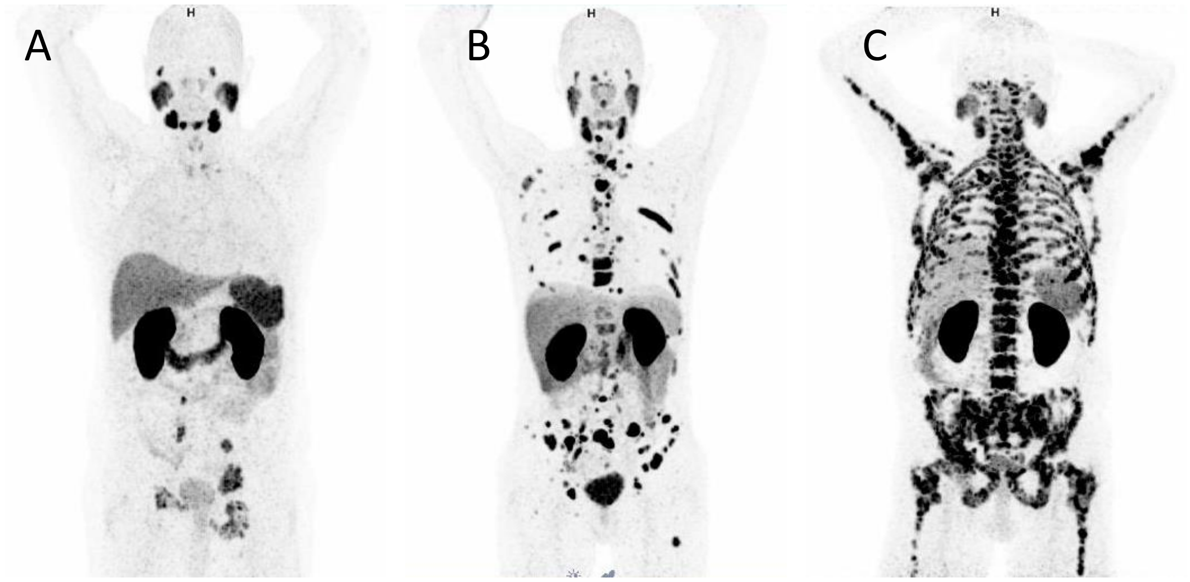

Supplementary Figure S1 Representative examples of subgroups based on the visual tumor burden assessed on ^{68}Ga-PSMA PET/CT at baseline. Maximum intensity projections (MIPs) of patients with A – low, B – moderate and C – high tumor load.

	SUVmax						*p**	MV (cm³)						*p**
	**baseline**			**follow-up**				**baseline**			**follow-up**			
	Mean	Min	Max	Mean	Min	Max		Mean	Min	Max	Mean	Min	Max	
right PG	13,9	4,8	25,3	11,5	5,3	20,9	*n.s.*	31,0	16,5	55,8	27,2	14,8	58,1	< *0.01*
left PG	14,6	4,9	29,7	12,2	5,0	22,4	*n.s.*	31,2	17,9	56,0	27,8	16,2	56,1	< *0.01*
right SMG	16,9	11,3	25,7	14,0	5,6	28,8	*n.s.*	11,4	5,7	16,8	10,9	4,6	18,1	< *0.05*
left SMG	18,1	9,2	30,6	14,2	6,0	28,7	< *0.05.*	11,7	7,1	17,8	11,2	8,0	18,0	< *0.05*

Supplementary Table S4 SUVmax and metabolic volume (MV) of the salivary glands on ^{68}Ga-PSMA PET/CT at baseline and follow-up of the Tandem-cohort: SUVmax showed a tendency to lower values, MV of all salivary glands declined significantly. (PG = parotid gland; SMG = submandibular gland) **Wilcoxon test*

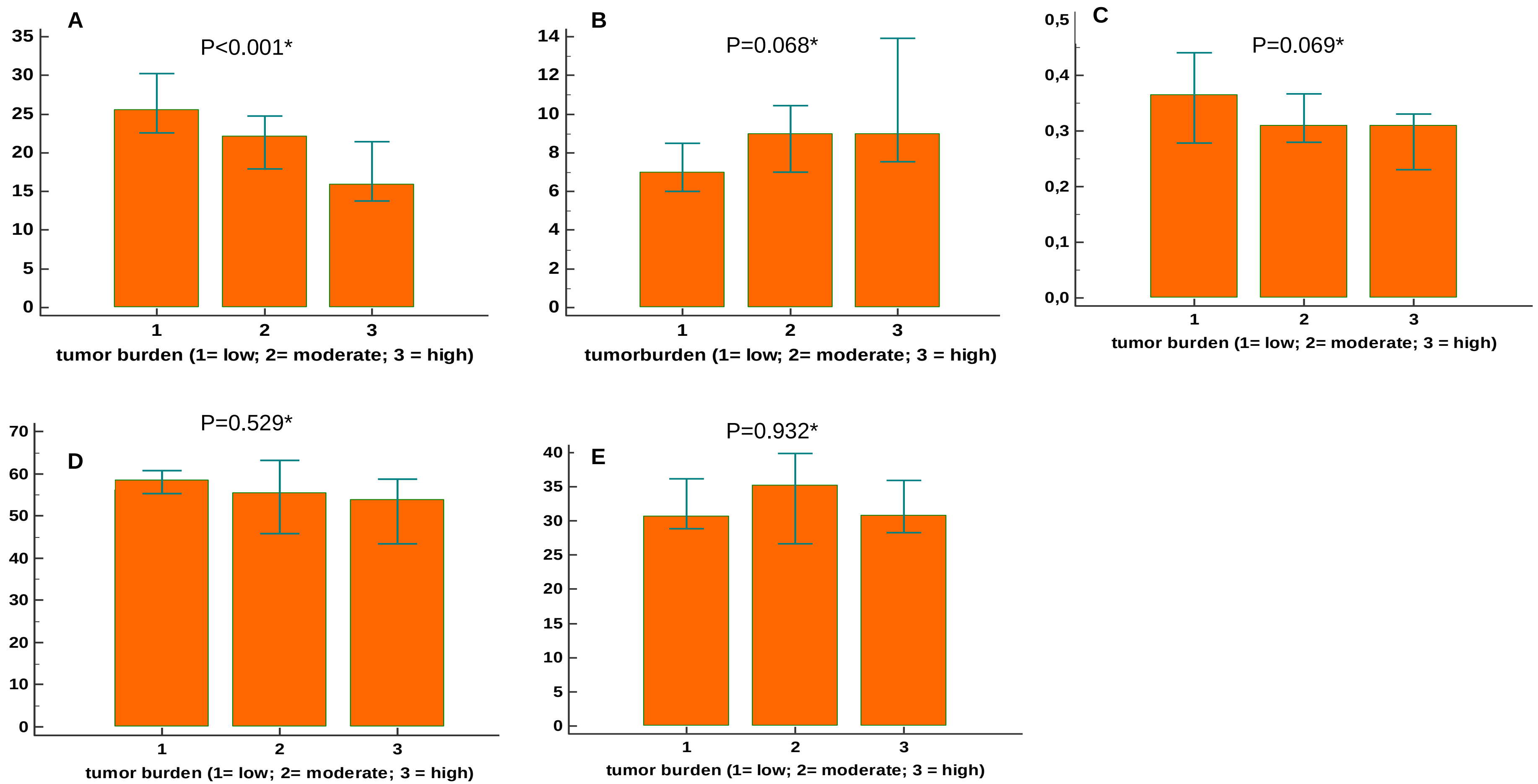

Supplementary Figure S2 Influence of tumor burden assessed on baseline PSMA PET/CT on A: SUVmax of SG at baseline; B: sXI at follow-up; C: Umax of PG at follow-up; D: EF of PG at follow-up; E: MV of PG at follow-up. (Umax= maximum uptake; EF= excretion fraction; MV= metabolic volume; PG = parotid gland; SMG = submandibular gland); * *Kruskal-Wallis test*

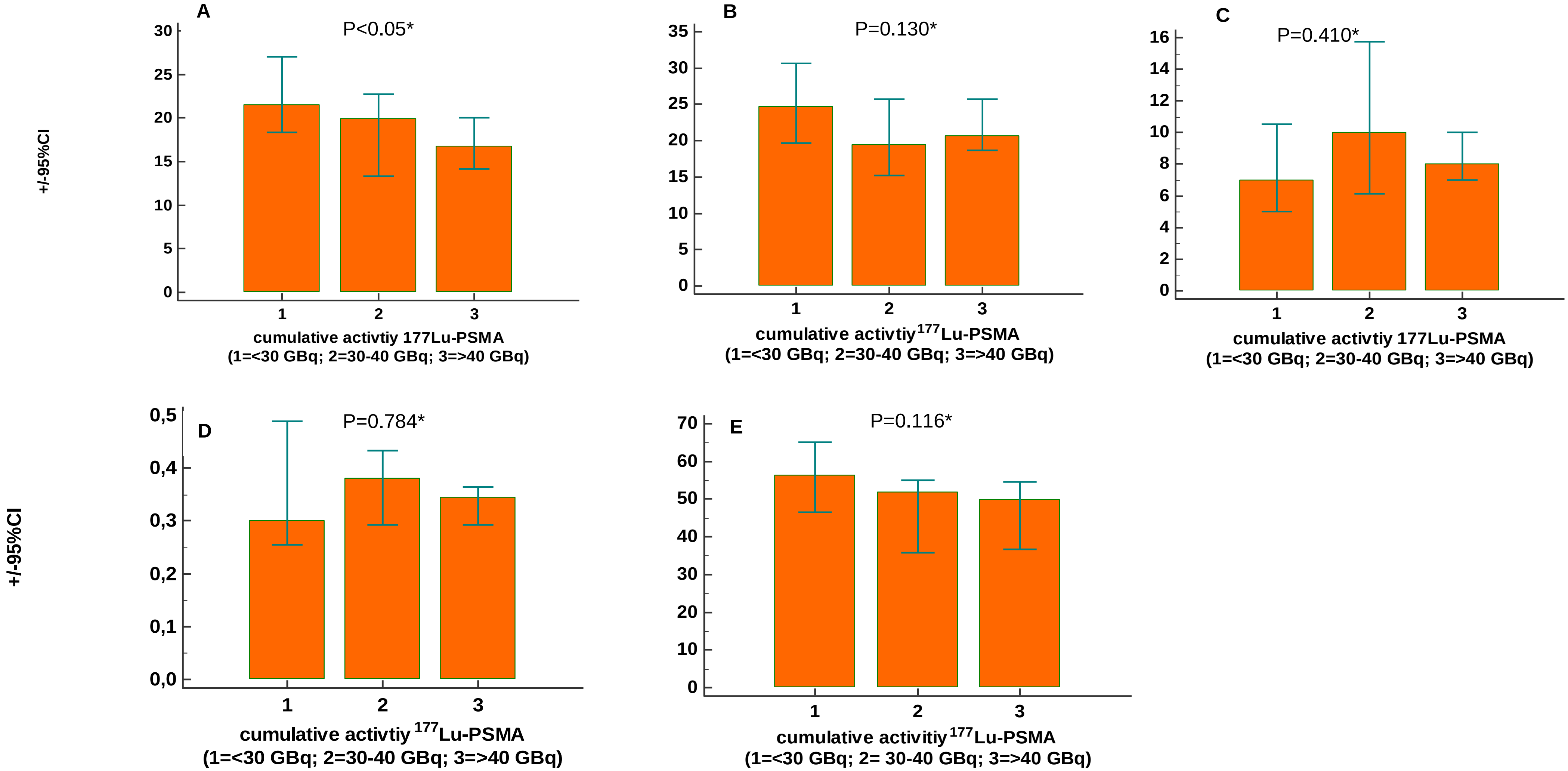

Supplementary Figure S3 Influence of the cumulative dose of ^{177}Lu-PSMA-I&T/-617 on A: SUVmax of SG at follow-up; B: MV of SG at follow-up; C: sXI score at follow-up; D: Umax of PG at follow-up; E: EF of PG at follow-up (Umax= maximum uptake; EF= excretion fraction; MV= metabolic volume; PG = parotid gland; SMG = submandibular gland); * *Kruskal-Wallis test*
